# First Complete Genome Sequence of Chronic Bee Paralysis Virus Isolated from Honey Bees (*Apis mellifera*) in China

**DOI:** 10.1128/genomeA.00618-16

**Published:** 2016-08-04

**Authors:** Beibei Li, Chunsheng Hou, Shuai Deng, Xuefeng Zhang, Yanna Chu, Chunying Yuan, Qingyun Diao

**Affiliations:** a Institute of Apicultural Research, Chinese Academy of Agricultural Sciences, Beijing, People’s Republic of China; b Key Laboratory of Pollinating Insect Biology, Ministry of Agriculture, Beijing, People’s Republic of China; c Graduate School of the Chinese Academy of Agricultural Sciences, Beijing, People’s Republic of China; d Guangdong Entomological Institute, Guangzhou, People’s Republic of China; e The Original Bee Breeding Farm of Liaoning Province, Xingcheng, People’s Republic of China

## Abstract

Chronic bee paralysis virus (CBPV) is a serious viral disease affecting adult bees. We report here the complete genome sequence of CBPV, which was isolated from a honey bee colony with the symptom of severe crawling. The genome of CBPV consists of two segments, RNA 1 and RNA 2, containing respective overlapping fragments.

## GENOME ANNOUNCEMENT

Chronic bee paralysis virus (CBPV) is the causal agent of adult bee paralysis, which results in severe losses of worker bees (1, 2). Although CBPV is considered a positive single-strand RNA virus, it has not been classified to any family. The morphology of virus particles and structural organization of RNA genome are distinctive profiles compared with those of other honey bee RNA viruses (3). The CBPV was first described in 1963 and induces two types of significantly different disease symptoms (1). One is abnormal trembling of the body and wings of bees, which frequently crawl in front of bee hives. Infected bees show bloated abdomens as a significant symptom (4). The other is hairless black bright disease. At the beginning, these bees can fly, and then they become flightless, hairless, and eventually die after a few days (5). However, these symptoms are easily confused due to the similarities with other RNA virus infection (6). Although full-genome sequences of a few CBPV strains have been reported (3), little is known about Chinese isolates. The full-genome sequence of CBPV reported here is from adult bees of Anhui Province, which suffered from heavy crawling. The genomic RNA of CBPV was extracted from samples from crawling bee using the QIAamp viral RNA minikit (Qiagen, Germany), and cDNA was generated with the SuperScript reverse transcriptase (Invitrogen, USA) and then sequenced by Shanghai Sangon Bio-Tech (Shanghai, China). The genome was assembled using Vector NTI Advance 11 software.

This is the first full-length sequence of CBPV reported in China, and the genome analysis of this Chinese isolate of CBPV showed that it consists of two segments, RNA 1 and RNA 2. RNA 1 is 3,657 nucleotides (nt) in length and contains a large open reading frame (ORF), which forms three small overlapping ORFs, including a putative viral RNA-dependent RNA polymerase (RdRp). RNA 2 contains 2,267 nt and shares 95% and 97% homology at the nucleotide level with French (accession no. EU122232) and Japanese (accession no. AB682803) strains. RNA 1 and RNA 2 of the CBPV genome differ significantly from those of other regions of the world at the amino acid level (7), although the genome structure of these two segments was similar to those genomes (3). This genome sequence will provide insights into better understanding of the epidemic and phylogeny of currently isolated CBPV. Also, it can lay the foundation for studying the infection mechanism and why it is at a high prevalence in China, especially in the spring.

### Nucleotide sequence accession numbers.

The genomic sequence of the chronic bee paralysis virus has been deposited in the GenBank under accession numbers KX168412 and KX168413.
